# Supplementary material for: Incidence and predictors of mortality among TB-HIV co-infected individuals on anti-tuberculosis and anti-retroviral dual therapy in Northwest Ethiopia: A retrospective cohort study
Source: PLoS One. 2025 Nov 26;20(11):e0337612. doi: 10.1371/journal.pone.0337612 (PMC12654907; doi:10.1371/journal.pone.0337612)
Supplement: S1 File — (DOCX) [file pone.0337612.s001.docx]

## 8.2 Annexe 2 Data extraction check list

**Part I. Study subject’s baseline information (to be filled from ART clinic intake form)**

| **Section one: socio-demographic characteristics of patients registration card** | | | |
| --- | --- | --- | --- |
| **S No.** | **Question /variables** | **Code and response** | **Skip** |
| **1.1.** | **Age of the patient** | **_______years** |  |
| **1.2.** | **Sex** | 1. **Male 2. Female** |  |
| **1.3.** | **Religion** | 1. **Orthodox 3.Muslim**   **2. Protestant 4.Catholic**   1. **Others (specific)__________** |  |
| **1.4.** | **Marital Status** | 1. **Married 3. Never married** 2. **Widowed 4. Divorced** 3. **Separated** |  |
| **1.5.** | **Ethnicity** | 1. **Amhara 3. Oromo** 2. **Tigre 4. Awi** 3. **Others (specify)______** |  |
| **1.6.** | **Place of residence** | 1. **Rural 2. Urban** |  |
| **1.7.** | **Educational status** | 1. **No education 3. Primary** 2. **Secondary 4. Tertiary**   **5.Others (specify)______** |  |
| **1.8.** | **Occupation** | **1.Merchant 3.Gov. Employee**  **2. Non-Gov. employed 4. Day Laborer**   1. **On Job seeking** 2. **Other please specify_______** |  |

| **Section two: Base line Clinical ,laboratory and ART information skip to;** | | | |
| --- | --- | --- | --- |
| **2.1.** | **WHO staging** | **1.Stage I**  **2.Stage II**  **3.Stage III**  **4.Stage IV** |  |
| **2.2.** | **CD4+ count dl/mm3**  **Date-----/-----/-----** | **_______________________** |  |
| **2.3.** | **Base line weight/kg** | **______________________** |  |
| **2.4.** | **ART eligibility criteria** | 1. **WHO clinical stage** 2. **Immunological/CD4Count** 3. **Both clinical and immunologic** |  |
| **2.5** | **Side effect during follow up** | 1. **Yes** 2. **No** |  |
| **2.6** | **Base line drug regimen type** | **1.AZT-based**  **2.D4T-based**  **3.TDF-based** |  |
| **2.7.** | **Baseline Hemoglobin(gm/dl)** | **_____________________________** |  |
| **2.8** | **Baseline functional status** | 1. **Working** 2. **Ambulatory** 3. **Bedridden** |  |
| **2.9** | **TB type** | 1. **Pulmonary TB** 2. **Extra-pulmonary TB** |  |
| **2.10** | **Substance use** | **1) Yes**  **2) no** |  |

| **Section three: Patient’s follow up information (to be filled from ART follow up form).Please document the current or the recent results** | | | **Skip** |
| --- | --- | --- | --- |
| **No.** | **Question/variables** | **Response and coded** |  |
| **3.1.** | **Date confirmed HIV +** | **Date -------/-----/-----** |  |
| **3.2.** | **Eligible date of ART** | **Date -------/-----/-----** |  |
| **3.3** | **Latest follow up date** | **Date -------/-----/-----** |  |
| **3.4.** | **Duration in months since**  **initiation of ART** | **____________in month** |  |
| **3.5.** | **recent weight in Kg** | **______Date -------/-----/-----** |  |
| **3.6.** | **recent functional status** | **1. Working 2. Ambulatory 3. Bedridden** |  |
| **3.7.** | **recent WHO staging** | 1. **Stage I 2. Stage II 3. Stage III 4.Stage IV** |  |
| **3.8.** | **Tb screened recently** | 1. **On treatment 2. Positive 3. Negative** |  |
| **3.9.** | **recent Tb prophylaxis** | 1. **No 2. Yes** |  |
| **3.10.** | **recent Tb treatment** | 1. **No 2. Yes** |  |
| **3.11.** | **Currently on cotrmoxazole** | 1. **Not given 2. Given** |  |
| **3.12.** | **Recent ARV adherence** | 1. **Good 2. Fair 3. Poor** |  |
| **3.13** | **Presence of treatment failure** | 1. **Yes** 2. **No** |  |
| **3.14** | **Type of treatment failure** | **1.Virologic failure**  **2.Immunologic failure** |  |
| **3.15.** | **recent CD4 count** | **_______Date--------/-------/------** |  |
| **3.16.** | **Recent Hgb (gm/dl)** | **_______Date--------/-------/------** |  |
| **3.17.** | **outcome of the patients** | 1. **Alive Date---------/------/-------** 2. **Died Date---------/--------/----** 3. **LOTL Date--------/-------/------** 4. **Transfer out Date-------/------/------** |  |
