# Supplementary material for: Incidence and predictors of mortality among TB-HIV co-infected individuals on anti-tuberculosis and anti-retroviral dual therapy in Northwest Ethiopia: A retrospective cohort study
Source: PLoS One. 2025 Nov 26;20(11):e0337612. doi: 10.1371/journal.pone.0337612 (PMC12654907; doi:10.1371/journal.pone.0337612)
Supplement: Attachment — Submitted filename: Comments to the author.docx [file pone.0337612.s002.docx]

Comments to the author

There are typographical and grammatical errors that need to be corrected throughout the document

The retrospective nature of the study might pose incomplete registration and losing of important variables. Was the registry you accessed were complete for all variables , how do you manage incomplete data

In your study being male were an important predictor mortality. What is the implication behind this finding

Abbreviation is not recommended in the abstract section of an article, please remove any abbreviations in the abstract

In your finding you reported that the ‘The mortality rate among TB-HIV co-infected patients at

Debre Markos Comprehensive Specialized Hospital was high’ what is your reference to say this number is big

In survival analysis, the median survival time is the preferred statistical test, reporting mean might be misleading

In your survival time description, there is no indications that censored observations were appropriately counted, which is critical in survival analysis
