# Supplementary material for: Incidence and predictors of mortality among TB-HIV co-infected individuals on anti-tuberculosis and anti-retroviral dual therapy in Northwest Ethiopia: A retrospective cohort study
Source: PLoS One. 2025 Nov 26;20(11):e0337612. doi: 10.1371/journal.pone.0337612 (PMC12654907; doi:10.1371/journal.pone.0337612)
Supplement: Attachment — Submitted filename: TB-HIV Comments.docx [file pone.0337612.s003.docx]

The tittle of the paper: Incidence and Predictors of Mortality among TB-HIV Co-infected Individuals on Antituberculosis and Anti-retroviral Dual Therapy in Northwest Ethiopia: A Retrospective

1. Give proper format of Abstract. Reframe it properly.
2. The manuscript needs extensive revision for language and grammar. Avoid using personal pronouns.
3. Fig 1, Fig 2 & Fig 3 needs proper explanation.
4. It is recommended to attach the questionnaire format.
5. The literature review may be rewritten to highlight comparisons with existing approaches.
6. Explain the proposed method thoroughly, incorporating the scenario to illustrate the work done.
7. Suggested to increase the data size.
8. Results section can be added and the practical limitation in the proposed methodology should be discussed in the results section.
9. It would be beneficial to include a graphical representation of the table for better understanding.
10. Authors are suggested to provide a comparative analysis to find the merit and demerit of existing and proposed methods.
11. Follow the format for references.
